# Inherited and Environmental Factors Influence Human Monocyte Heterogeneity

**DOI:** 10.3389/fimmu.2019.02581

**Published:** 2019-11-07

**Authors:** Amit A. Patel, Simon Yona

**Affiliations:** ^1^Division of Medicine, University College London, University of London, London, United Kingdom; ^2^The Institute of Dental Sciences, Hebrew University, Jerusalem, Israel

**Keywords:** monocyte, macrophage, inflammation, sex, age, diet, exercise, sleep

## Abstract

Blood monocytes develop in the bone marrow before being released into the peripheral circulation. The circulating monocyte pool is composed of multiple subsets, each with specialized functions. These cells are recruited to repopulate resident monocyte-derived cells in the periphery and also to sites of injury. Several extrinsic factors influence the function and quantity of monocytes in the blood. Here, we outline the impact of sex, ethnicity, age, sleep, diet, and exercise on monocyte subsets and their function, highlighting that steady state is not a single physiological condition. A clearer understanding of the relationship between these factors and the immune system may allow for improved therapeutic strategies.

## Introduction

Adult blood leukocytes can be separated into two broad categories: lymphoid or myeloid. The lymphoid lineage consists of T, B, innate lymphoid and natural killer (NK) cells, while the myeloid compartment comprises of functionally and morphologically discrete cell types, including mononuclear phagocytes (monocytes and dendritic cells), granulocytes (neutrophils, basophils, and eosinophils), and platelets. Injured or infected tissue releases chemoattractants that rapidly recruit these myeloid cells to the site of injury. Once at the inflamed site, these cells coordinate and carry out key effector functions. Nearly 100 years ago, Alexander Maximow suggested that hematopoiesis was an extremely organized stepwise process arising from a common precursor cell (1). Indeed, hemopoietic stem cells that reside in the bone marrow undergo multiple differentiation stages, becoming progressively more restricted and eventually give rise to a heterogeneous population of leukocytes. Commitment to the myeloid lineage downstream of the common myeloid progenitor (CMP) (2) results in the generation of erythrocytes, platelets, granulocytes, monocytes, and dendritic cells. Nevertheless, several extrinsic factors influence leukocyte generation. Here, we will outline several studies that highlight how age, ethnicity, diet, exercise, sleep, and sex modulate human monocyte numbers under healthy homeostasis.

Circulating blood monocytes were initially believed to be a single population of cells with the potential to repopulate terminally differentiated resident mononuclear phagocytes in the periphery (3). In addition, blood monocytes act as an emergency squad recruited to sites of injury where they perform pro-inflammatory and pro-resolving functions (4–8). Blood monocytes were initially defined by their morphology. Later, with the introduction of flow cytometry, monocytes were shown to express high levels of the lipopolysaccharide (LPS) binding protein receptor, CD14 (9). These CD14^+^ monocytes were subsequently discovered to be a heterogeneous population that could be further divided into discrete subsets based on CD14 and CD16 (FcγRIII) expression in humans (10). Monocyte heterogeneity was later observed to be conserved among other species (11). Human CD14^+^ CD16^−^ monocytes, also known as classical monocytes, are analogous to the murine Ly6C^Hi^ CX_3_CR1^int^ classical monocytes. Intermediate and non-classical monocytes are identified as CD14^+^, CD16^+^, and CD14^lo^CD16^+^ cells, respectively, where the latter subset mirror Ly6C^Low^ CX_3_CR1^Hi^ non-classical monocytes in mice (12, 13). The expression of several membrane adhesion molecules and chemokine receptors can also be used to discriminate between these monocyte subsets (13–15).

**Figure 1:**
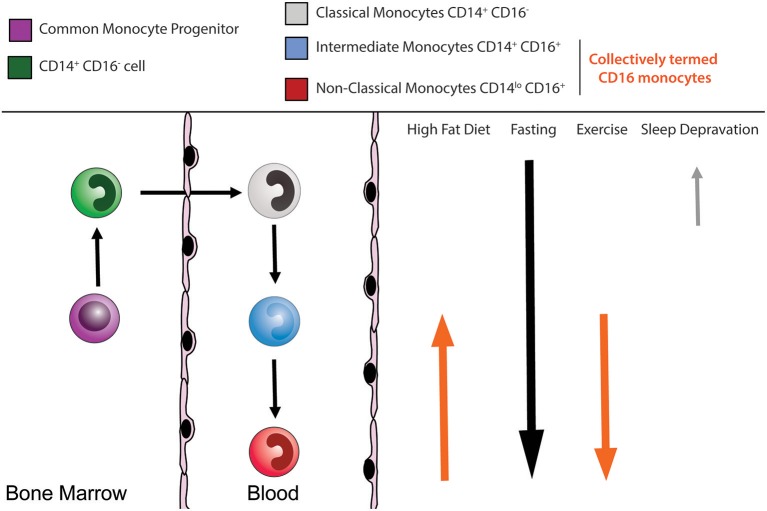
Human monocyte subsets. Circulating monocytes arise in the bone marrow from a common monocyte progenitor (cMoP) before being released into the peripheral circulation. The circulating monocyte pool is composed of multiple subsets. Human CD14^+^ CD16^−^ classical monocytes (gray), CD14^+^ CD16^+^ intermediate monocytes (blue), and CD14^lo^CD16^+^ non-classical monocytes (red). Several external lifestyle factors can impact on these monocyte subsets.

Blood monocytes begin their life in the bone marrow, following a similar developmental fate to dendritic cells where they both arise from the macrophage/dendritic cell precursor (MDP) (16). In mice, the MDP was initially proposed to give rise to monocytes and dendritic cells but not granulocytes (17). Downstream of the MDP, the common monocyte progenitor (cMoP) was identified, which gives rise exclusively to classical monocytes (18). The human equivalent of the murine cMoP was recently identified within the bone marrow granulocyte-monocyte progenitors (GMP) fraction (19). This human cMoP gives rise directly to pre-monocytes, and subsequently, monocytes. Investigations into the developmental stages between the cMoP and mature monocytes uncovered in mice a Ly6C^+^ CXCR4^+^ monocyte subset that resides in the bone marrow termed pre-monocytes (20). These pre-monocytes show proliferative potential and downregulate CXCR4 upon entry into the circulation (20). The egression of murine classical monocytes from the bone marrow follows a circadian rhythm, regulated in part by CXCR4 (20) and the circadian gene *Bmal1* (21). It is widely accepted that classical and non-classical monocyte subsets are related developmentally, with classical monocytes having the potential to give rise to non-classical monocytes over time (14, 22–27) (Figure 1). While recent advances demonstrate monocyte development to be a highly regulated process under steady state, here we summarize the influence of inherited traits and lifestyle choices have on human monocyte homeostasis.

## Lifestyle and Genetic Factors Affecting Monocytes

Our knowledge concerning the development and function of monocytes and monocyte-derived cells during pathology continues to expand. It is also necessary to appreciate the behavior of these cells under healthy physiological conditions. However, healthy homeostasis is not a solitary state, rather several factors—often overlooked—including sex, diet, exercise, and age influence the immune system. Whether genetic traits prevail over environmental cues regarding the immune response remains a matter of debate (28, 29).

The earliest accounts of the cell originate with Robert Hooke's seminal observations in 1665 (30). Cellular analysis began with microscopy, then progressed to flow cytometry and is currently enjoying a renaissance in the form of single-cell RNA analysis. Every advancing stride has led to a greater appreciation regarding the complexity and diversity of monocytes, their subsets, and function (31–34). Here, we consider if and how, lifestyle choices imprint on this diversity. This review will focus on our current understanding of human monocyte adaptations observed due to genetic and environmental factors; for a comprehensive review on monocyte biology, see Guilliams et al. (5) and Jakubzick et al. (6).

As a word of caution, the monocyte nomenclature was recently codified by Ziegler-Heitbrock et al. and approved by the International Union of Immunological Societies (35, 36). Nevertheless, complexity remains an issue within the historical literature and is further confounded by subsets conveyed sometimes as a proportion of total monocytes or in absolute numbers. Here, we have retold studies as originally described to avoid confusion.

## Sex

Several physiological differences exist between the sexes, the most apparent being their role in reproduction. Another obvious difference is hormone concentration. Over three quarters of patients with autoimmune disease are female (37). Systemic lupus erythematosus (SLE), Sjögren syndrome, fibromyalgia, and rheumatoid arthritis afflict females more than males (38, 39), whereas ankylosing spondylitis, vasculitis, and Goodpasture syndrome predominantly occur in males (40). This sexual dimorphism of autoimmune-driven disease begs the question, do male and female immune systems differ?

Under physiological conditions, monocyte counts have consistently been reported to be elevated in males at all stages of life (41–43). This difference is most profound during adolescence (44). Curiously, one study examining over 400 individuals reported that monocytes are higher in Caucasian men than those in women; this difference was absent in the Afro Caribbean population (41). The proportion of non-classical monocytes has also been reported to be different amongst men and women (45). These differences in monocyte subsets may be attributed to the effect of estrogen and other sex hormones. To test this, researchers have turned to the menstrual cycle and menopause. During the menstrual cycle, 17 β-estradiol, and progesterone concentrations are consistently increased during the luteal phase in comparison to the follicular phase. Around 40 years ago, an elevated monocyte count was reported during the luteal phase (46). With our growing knowledge on monocytes, it would be worthwhile to revisit the impact of the menstrual cycle on monocyte subpopulations, especially as the identification of monocytes subsets remains inconsistent throughout the literature. Interestingly, postmenopausal women exhibit lower concentrations of estrogen and tend to have an increased blood monocyte count; nonetheless, following estrogen replacement therapy, monocyte numbers were restored to levels seen in younger females (47). These data suggest an increase in estrogen decreases monocyte numbers, supporting the observation that males tend to have a higher monocyte count. An exception to this would be the increase in monocytes observed during the luteal phase of the menstrual cycle. Together, this may suggest other endogenous factors affect monocyte composition.

Sex differences may become conspicuous in the disease setting. Both healthy males and females have equivalent number of intermediate monocytes; however, only male sarcoidosis patients exhibit an elevated number of intermediate monocytes compared to female sarcoidosis patients who had equivalent numbers to healthy female controls (48).

Sexual dimorphism has been reported in monocyte cytotoxic activity (49) and cytokine production. Male monocytes produce more inflammatory cytokines than females when stimulated with LPS (42); however, female sex hormones were not responsible for this effect as demonstrated *in vivo* by oral contraceptive use (50). It is important to note that these studies used LPS. PBMCs co-cultured with estrogen had altered expression of TLR3, TLR7, TLR8, TLR9, but not TLR2 and the LPS receptor, TLR4 (51). Therefore, the impact of estrogen on monocyte function may only become apparent in response to particular stimuli. These diverse responses on monocyte function between the sexes are discussed in detail (52). Further studies will help define the cytokines and/or hormones responsible for the divergence observed in monocyte count and function between males and females.

## Ethnicity

Ethnic diversity is reflected in disease susceptibility across different human populations (53). This has been identified in patients with tuberculosis (TB) infection (54), autoimmune hepatitis (55), and systemic lupus erythematosus (SLE) (56). A groundbreaking study by Nédélec et al. proposes that different environmental pressures on our immune system may explain why African ancestry is associated with a stronger inflammatory response compared to Europeans (57).

Ancestry has been shown to influence leukocyte counts, including neutrophils, lymphocytes, eosinophils, and monocytes (41, 58, 59). A trans-ethnic meta-analysis study revealed that those of a European ancestry tend to have a higher monocyte count compared to African-American and Japanese individuals (59). As expected, the interplay of ethnicity and other variables is likely to influence monocyte count. Caucasian males were observed to have a significantly higher count compared to African males, while no difference was noted for females (41), demonstrating the interaction of sex and ethnicity.

Regarding individual monocyte subsets, recently, a single study has shown that Caucasian populations tend to have a higher percentage of classical monocytes and conversely a lower percentage of non-classical monocytes than those sampled from an African population (58). In this study, Caucasians had a trend for increased CX_3_CR1 expression; this could explain the difference in monocyte proportions. CX_3_CR1 is involved in non-classical monocyte retention to the endothelium (60, 61), as well as a survival factor for this monocyte population (62); therefore, this increased expression might result in lower representation of free circulating non-classical monocytes due to their increased adherence. Of note, ethnic differences are possibly reflected in monocyte function, as monocyte-derived cells from Filipino, Chinese, and non-Hispanic whites challenged with *Mycobacterium tuberculosis* produced varying quantities of cytokines from 137 volunteers (>44 from each background) (63). As ethnicity can influence the immune response, this may implicate the need for a more personalized take regarding therapeutics.

## Diet

Diet varies from individual to individual—from what they eat to the quantity and frequency. A balanced diet is a basic requirement for a healthy lifestyle. This fine balance goes awry during surplus calorie intake, which contributes to many diseases, including atherosclerosis, type 2 diabetes, and non-alcoholic fatty liver disease (NAFLD). These western diet-related diseases are associated with systemic chronic inflammation (64).

Monocytes play a central role in several diet-related pathologies. In recent years, it has become evident that a high-fat western diet triggers a number of functional modifications in monocytes. Mice fed a western diet for 4 weeks led to an elevation in circulating and splenic monocytes compared to those fed on a standard chow diet (65). Further examination into how a western diet prompts myelopoiesis was described to be due to an increase in GMP in the bone marrow that also functionally reprograms myeloid cells through NLRP3. Upon reverting to a chow diet, monocyte numbers returned to baseline, although an increased activation status became imprinted in classical monocytes. Collectively, these data suggest that diet leads to innate immune training within the monocyte pool in mice. In a human study, 3 h after the consumption of a high-fat (McDonald's) meal resulted in an increased monocyte count, specifically an elevation in non-classical monocytes (66). Similarly, this observation is consistent with a study where CD16^+^ monocytes (intermediate and non-classical) positively correlated with increased body weight (67). Interestingly, in these subjects, dietary intervention or gastric bypass surgery resulted in a decrease in the absolute number and percentage of these cells (67). Immediately following a high-fat meal, Khan et al. demonstrated a higher percentage of lipid-laden monocytes (66). These foamy monocytes increase their expression of CD11c, which is thought to lead to monocyte arrest *via* vascular cell adhesion protein (VCAM-1). While these are short-term effects, recurrent chronic exposure of a high-fat diet may lead to diet-related diseases.

Although a high-fat dietary intake increases peripheral monocyte numbers, the opposite is true in fasted individuals (68). Dietary restriction for 19 h in humans or 4 h in mice led to a significant reduction in both circulating classical and non-classical monocytes. This reduction in blood monocytes was due to the inhibition of monocyte egress from the bone marrow. This elegant study from the Merad group demonstrated that carbohydrate and protein consumption rescues monocyte numbers *via* the liver activated protein kinase-peroxisome proliferator-activated receptor alpha (AMPK-PPARα) pathway that regulates the monocyte chemoattractant protein, CCL2 (68). Furthermore, dietary restriction during experimental autoimmune encephalomyelitis (EAE), a mouse model of multiple sclerosis, improved disease outcome and reduced myeloid cell infiltrate compared to animals with access to food *ad libitum* (68, 69). Similar findings have been observed in humans, where fasting diets have shown to improve the quality of life for patients with multiple sclerosis (69, 70).

In an experimental setting, a single macronutrient or micronutrient alters the monocyte composition and function. It is more realistic that it is a combination of several dietary nutrients in varying amounts that will alter the phenotype of monocytes.

## Sleep/Wake Cycle

Cortisol is the archetypical neuroendocrine anti-inflammatory hormone that regulates immune cell gene expression (71, 72). Cortisol follows a diurnal pattern where it peaks 30 min after waking in the morning and falls throughout the day. Cortisol is the endogenous member of the glucocorticoid family of immunosuppressive and anti-inflammatory drugs that acts on many leukocytes, including monocytes, where they induce a rapid decrease in circulating monocytes (73). Therefore, it would be interesting to know if monocytes also follow a diurnal pattern.

Meuret et al. performed to the best of our knowledge one of the earliest studies on monocyte kinetics. Meuret and colleagues observed a monocyte cycle occurring around every 5 days in humans. They proposed this due to a homeostatic loop within the bone marrow with transit time being the prevailing factor (74). Recently, we observed a population of CD14^+^ CD16^−^ cells resembling classical monocytes that reside in the human bone marrow (14); these cells exhibit a postmitotic dwell period of ~1.5–2 days before being released into the circulation (14, 75). It is possible that this maturation period, in addition to the time taken to differentiate into these cells, and the regulatory signals account for this monocyte cycle.

While long-term monocyte oscillations have been described, diurnal patterns are also present in both mice (20, 21) and humans, where monocyte numbers decrease during sleep and begin to gradually rise upon waking (76, 77). Cuesta et al. were able to stratify individuals into two categories, one where monocyte numbers peak during the morning, and another group where the monocyte count peaked in the evening (77). The reason behind the existence of these two groups remains unclear—all subjects except one were male, of a similar age who maintained comparable levels of activity, equivalent calorie intake, and exposure to light. In mice, CXCR4 and *Bmal1* regulate the circadian rhythm of circulating monocytes (20, 21).

Functional changes such as cytokine production, surface membrane protein expression, and phagocytic ability have been reported to follow a diurnal pattern (77–80). Cuesta et al. conclude that cytokine production follows a bimodal rhythm, where monocytes are more responsive at night, whereas during the day, a higher production results from the increased numbers of monocytes (77). In addition, while TLR1, 2, 4, and 9 expression does not differ throughout the day, activation of these receptors results in the dampened expression of costimulatory molecules in the morning (78).

Sleep-deprived individuals who remain awake during the night gain a higher monocyte count compared to those who slept at this period (76). Congruent with these findings, mice with disrupted sleep also have an increase in peripheral blood classical monocytes (81). Uninterrupted sleep enables the release of hypocretin from the hypothalamus, which in turn regulates CSF1 production from bone marrow pre-neutrophils regulating monopoiesis (81). Although an increase in monocyte count occurs during sleep deprivation, a diurnal oscillation pattern remains (82). Furthermore, in night shift workers who are active at night, no phase shift was detected in monocyte numbers (77). Taken together, these studies establish that both internal and external body clocks influence monocyte behavior and emphasize the importance of appropriate time controls when conducting experiments.

Circannual or seasonal rhythms have also been detected in monocyte function. Monocyte counts remain stable throughout the year, yet phagocytosis, cytokine production, and prostaglandin metabolism vary (83, 84). Specifically, a higher proportion of monocytes phagocytose in winter compared to spring and autumn. In response to LPS, monocytes isolated during the autumnal period produce lower concentrations of both inflammatory [tumor necrosis factor (TNF)-α and interleukin (IL)-6] and anti-inflammatory (IL-10) cytokines. There are several possible explanations for the fluctuation in human monocyte function throughout the year; seasonal changes may be influenced by a myriad of factors including increased periods in crowded places (i.e., indoor contagion theory), reduced exposure to sunlight (vitamin D deficiency), or even temperature changes. Despite the reason for these changes, these seasonal changes may be relevant when performing long-term clinical trials and should be taken into consideration.

## Exercise

Over 120 years ago, Schulte described that exercise impacts the human immune system and induces leukocytosis (85). Therefore, the question arises, do monocytes fluctuate during exercise? Studies have reported a rise in intermediate and non-classical monocytes immediately following exercise (86), while others have described a change in classical monocytes (80), classical and non-classical monocytes (87), and even all three populations (88, 89). At first glance, this can appear confusing however; not all exercise is equal as the intensity, duration, and the type of exercise (aerobic or anaerobic) influence monocytes (90) and may account for these different findings. Maximal oxygen consumption, a readout of an individual's fitness, negatively correlated with the percentage change in intermediate monocytes (89). Therefore, the larger the maximal oxygen consumption, the smaller the percentage change in intermediate monocytes. The majority of non-classical monocytes are constantly crawling on the endothelium in an LFA1-dependent manner (60, 61). This may be overcome during intense exercise by the increase in shear blood flow. Patrolling monocytes that previously were crawling on endothelial cells are now able to be isolated, providing a more genuine picture of blood monocyte subsets. While these effects are transient, long-term alterations in monocyte composition have also been observed following exercise. A 12-weeks exercise regime reduced the percentage of CD16^+^ monocytes (91). This decline in intermediate monocytes could be associated with fat loss (67), as described above. In addition, TNF-α production was significantly reduced following this 12-weeks exercise program, while monocyte phagocytosis increased, suggesting that long-term exercise may promote a more anti-inflammatory monocyte while improving its phagocytic capacity.

## Age

At birth, monocytes are three times higher compared with those of adults (92). Christensen and colleagues analyzed over 63,000 hospital records and found that monocyte counts increase linearly with gestational age (93). This monocyte expansion continued into the first 2 weeks after birth. As an organism ages, so does its immune system. The term inflamm-aging was coined by Franceschi 20 years ago (94) to describe the progressive increase in the organism's proinflammatory status as it matures. Inflamm-aging includes adaptive immunity, immunosenescence, and dysregulation of the innate immune response. While some studies have reported no changes in the mononuclear phagocyte count in different age cohorts (43, 95), others have noticed a decrease in dendritic cells (96) and an increase in monocyte subsets in the older aged cohort (45, 96–99), particularly in intermediate and non-classical monocytes. Plasma CCL2 is elevated in older individuals (99), which may result in monocyte mobilization from the bone marrow, resetting the dynamic equilibrium of blood monocyte subsets. However, in advanced old age (81–100 years), fewer classical and intermediate monocytes have been detected (100).

Coincidently, CD16^+^ monocytes have been reported to expand in inflammatory conditions, also increased in older individuals. These monocytes, in particular, non-classical monocytes, produce higher basal levels of TNF-α and is thought to account for the increase in plasma TNF-α levels in aged individuals (45, 97, 101). A consequence of this inflammatory environment results in dysregulated innate immunity, such as impaired phagocytosis (45). The pharmacological blockade of TNF-α in aged mice improved bacterial clearance and returned classical monocytes to levels in young mice (102). Therefore, the accumulation of monocytes in the elderly may account for age-related inflammatory conditions. While plasma TNF-α changes with age, hormonal changes also occur. Therefore, it is important to consider age and environmental and genetic factors, as all interact with the immune system. There are as many “young” 85-year-olds running marathons as “old” sedentary 75-year-olds.

## Conclusion

Recently, new monocyte subsets have been described in mice (35, 103–105) and humans (32, 34). The identification of these new populations demonstrates how recent technological advances have changed the mononuclear phagocyte landscape. Future insight to these subsets will provide therapeutic strategies to enhance these cells when they are beneficial and block the detrimental effects. In the quest for novel therapeutics, it is critical to remember how sex and environmental and lifestyle choices lead to physiological variations within a leukocyte population as discussed here for monocytes. It is important to examine these variables from a holistic stance using defined objective criteria to avoid bias that may have crept into previous studies. Historically, research into the impact of lifestyle choices was performed on a limited cohort with certain subjective evaluations. The adoption of electronic health records will provide greater insight into how sex and environmental and lifestyle choices impact monocytes and additional leukocyte populations on a previously unimaginable scale.

## Author Contributions

All authors listed have made a substantial, direct and intellectual contribution to the work, and approved it for publication.

### Conflict of Interest

The authors declare that the research was conducted in the absence of any commercial or financial relationships that could be construed as a potential conflict of interest.
